# Molecular classification of cancer with the 92-gene assay in cytology and limited tissue samples

**DOI:** 10.18632/oncotarget.8449

**Published:** 2016-03-28

**Authors:** Elena F. Brachtel, Theresa N. Operaña, Peggy S. Sullivan, Sarah E. Kerr, Karen A. Cherkis, Brock E. Schroeder, Sarah M. Dry, Catherine A. Schnabel

**Affiliations:** ^1^ Department of Pathology, Massachusetts General Hospital, Boston, Massachusetts, USA; ^2^ Biotheranostics, Inc., San Diego, California, USA; ^3^ Department of Pathology and Laboratory Medicine, David Geffen School of Medicine, University of California Los Angeles, Los Angeles, California, USA; ^4^ Department of Laboratory Medicine and Pathology, Mayo Clinic, Rochester, Minnesota, USA

**Keywords:** gene expression profiling, biological markers, molecular targeted therapy, cytology, clinical oncology

## Abstract

**Background:**

Detailed molecular evaluation of cytology and limited tissue samples is increasingly becoming the standard for cancer care. Reproducible and accurate diagnostic approaches with reduced demands on cellularity are an ongoing unmet need. This study evaluated the performance of a 92-gene assay for molecular diagnosis of tumor type/subtype in cytology and limited tissue samples.

**Methods:**

Clinical validation of accuracy for the 92-gene assay in limited tissue samples such as cytology cell blocks, core biopsies and small excisions was conducted in a blinded multi-institutional study (N = 109, 48% metastatic, 53% grade II and III). Analytical success rate and diagnostic utility were evaluated in a consecutive series of 644 cytology cases submitted for clinical testing.

**Results:**

The 92-gene assay demonstrated 91% sensitivity (95% CI [0.84, 0.95]) for tumor classification, with high accuracy maintained irrespective of specimen type (100%, 92%, and 86% in FNA/cytology cell blocks, core biopsies, and small excisions, respectively; *p* = 0.26). The assay performed equally well for metastatic versus primary tumors (90% vs 93%, *p* = 0.73), and across histologic grades (100%, 90%, 89%, in grades I, II, and III, respectively; *p* = 0.75). In the clinical case series, a molecular diagnosis was reported in 87% of the 644 samples, identifying 23 different tumor types and allowing for additional mutational analysis in selected cases.

**Conclusions:**

These findings demonstrate high accuracy and analytical success rate of the 92-gene assay, supporting its utility in the molecular diagnosis of cancer for specimens with limited tissue.

## INTRODUCTION

**Figure 1:**
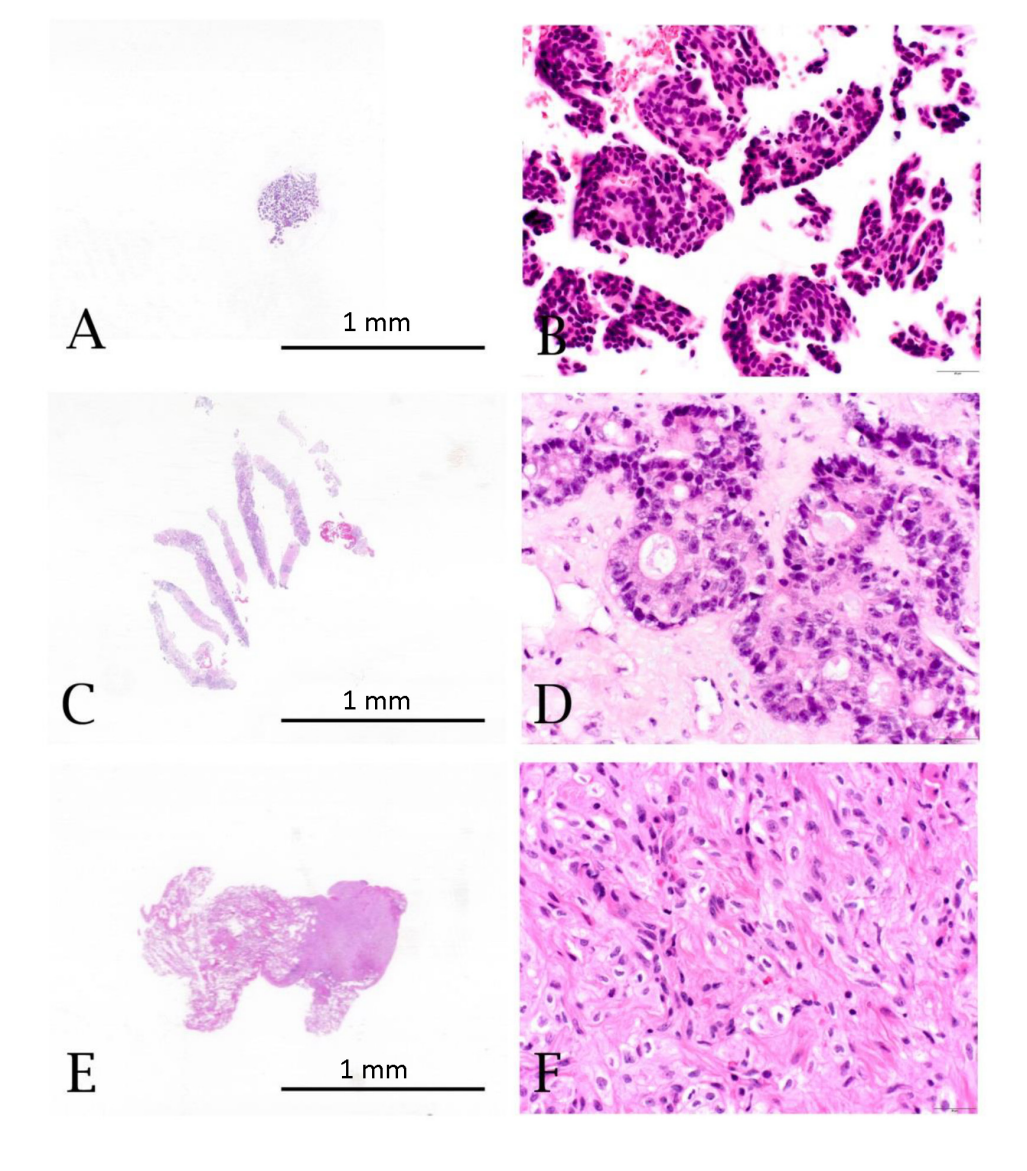
Hematoxylin and eosin stained specimens from limited tissue sample types **A.** & **B.** Fine needle aspirate (FNA)/cell block showing breast adenocarcinoma (10X and 400X). **C.** & **D.** Core biopsy showing colorectal adenocarcinoma metastatic to the liver (10X and 400X). **E.** & **F.** Small excisional biopsy showing renal cell carcinoma metastatic to the lung (10X and 400X).

While molecular testing in oncology continues to develop and validate new methodologies employing reduced cellularity, such as circulating tumor cells (CTCs) and cell-free tumor material, the current standard of care depends on appropriate management of tumor tissue biopsy specimens for diagnosis and ancillary studies [1, 2]. Often, cytology samples from fine needle aspirations (FNAs), fluids or core biopsies are the only tissue samples available for diagnosis and advanced molecular interrogation (Figure 1). As the tissue samples obtained by minimally invasive diagnostic procedures continue to decrease in size, the demand increases for detailed diagnostic evaluation and molecular testing using these limited tissue samples [3]. In addition to new sampling procedures such as endoscopically guided fine needle aspirations, new genomic technologies such as next generation sequencing (NGS) are being used by clinicians to simultaneously evaluate multiple genes/signaling pathways to identify actionable mutations for treatment planning [4]. Use of tissue conserving technologies will ensure that specimens are available for diagnostic and molecular analyses as oncology disease management evolves.

Limited tissue samples are small and often exhausted during routine histology and immunohistochemical studies. In response to continuing developments and in an effort to conserve tissue samples, molecular tests for diagnosis and targeted therapy approaches are being incorporated into clinical practice and molecular testing guidelines for various tumor types and subtypes [5, 6]. Several diagnostic assays for gene expression-based classification of tumors requiring minimal tissue are already in use [7, 8]. These molecular tools provide independent quantitative data that is standardized and complementary to routine morphology and immunohistochemistry (IHC) approaches [8–10]. Integrating gene expression profiling into diagnostic algorithms for samples that have limited cellular content or lack definitive morphological and immunohistochemical features allows the remaining tissue to be used for reflex biomarker testing [11]. The goal of these various approaches is to ensure accurate diagnosis and allocation of tissue from small samples for the appropriate ancillary studies.

The 92-gene assay is a molecular classifier that predicts tumor type and subtype through an algorithmic-based comparison of a tumor's gene expression profile to a reference database of known tumor types. The assay distinguishes between 28 main tumor types and 50 subtypes, which represents >95% of all solid tumors based on incidence [8, 12]. The assay employs a real-time RT-PCR, tissue-sparing platform, requiring approximately 300-500 non-necrotic tumor cells, that interrogates expression of transcription factors, plasma membrane proteins, and tissue specific tumor markers not commonly evaluated during routine IHC analyses to assess over 350 unique biological functions [13]. In a previously published validation study, the 92-gene assay demonstrated 87% (95% CI, 84-89%) overall accuracy, and notably, statistically similar performance in metastatic and high grade tumors [8]. In a head-to-head comparison vs IHC, the 92-gene assay demonstrated a statistically significant improvement in diagnostic accuracy in poorly-to-undifferentiated metastatic tumors (79% vs 69%; P = 0.019) [14]. Additionally, when the molecular diagnosis provided by the 92-gene assay was used to direct therapy in treatment-naïve patients with carcinoma of unknown primary, a 3.4 month increase in overall survival was observed when compared to a pre-specified historical cohort [15]. In the current study, the specific performance and analytical success rate of the 92-gene assay in tumors from limited tissue and cytology specimens were further characterized to assess its diagnostic utility within the current landscape of precision oncology.

## RESULTS

### Specimen and perfomance characteristics of clinical validation cohort

#### Specimen and patient characteristics for clinical validation cohort

**Figure 2:**
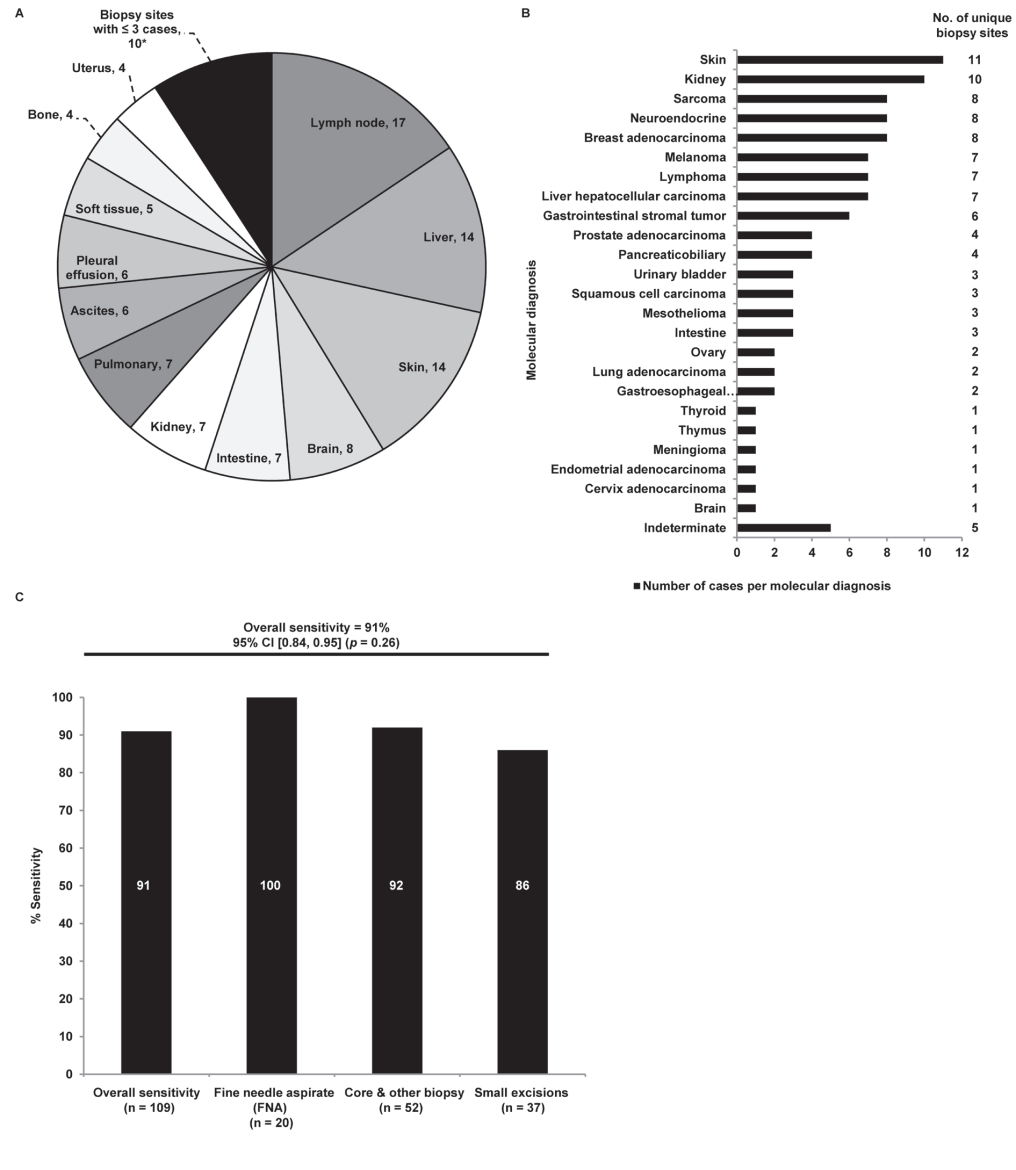
**A.** Distribution of biopsy sites from clinical validation cohort. *Biopsy sites with three or fewer cases were combined into a single category, which includes: Breast (3), Adrenal (2), Mediastinum (2), Prostate (2), Retroperitoneum (1). **B.** Distribution of molecular diagnoses as determined by the 92-gene assay in the limited tissue data set. Molecular diagnoses labeled on the y-axis. Number of unique biopsy sites for each molecular diagnosis is labeled to the right of the graph. **C.** Sensitivity of the 92-gene assay for each specimen type in the limited tissue data set (*p* = 0.26). Specimen type labeled along the x-axis. Sensitivity expressed as a percentage is shown by each column. Exact values per specimen type are shown in white. Overall sensitivity for the 92-gene assay was 91%.

Case characteristics for the clinical validation cohort (N = 109) are summarized in Table 1. Approximately half (48%) of all tumor specimens were from metastatic lesions. Tumors were primarily intermediate or high grade (I = 5%, II = 18%, III = 35%). Forty-two percent of cases were ungraded including metastatic lesions and primary cases in which tumor grading was not standard procedure during the diagnostic evaluation or not traditionally conducted in certain tumors such as pheochromocytomas and gastrointestinal stromal tumors. Specimens with 50% or less tumor content, greater than 20% fibrosis and 5-30% necrosis comprised 29%, 25%, and 36% of the cases analyzed, respectively. The biopsy types evaluated for this data set included FNA/ cell blocks (N = 20, 18%), core / other biopsies (N = 52, 48%), and small excisions (N = 37, 34%) (Table 1 and Figure 1). The cases were distributed among 17 biopsy sites and included 24 different tumor types (Figure 2A and 2B).

**Table 1:** Tumor characteristics for the clinical validation cohort (N=109)

Characteristic	N (%)
Gender	
Male	54 (49)
Female	55 (51)
Primary	57 (52)
Metastatic	52 (48)
Histologic Grade	
Grade I	5 (5)
Grade II	20 (18)
Grade III	38 (35)
Not graded	46 (42)
Tumor content	
≤ 50%	32 (29)
> 50%	77 (71)
Fibrosis	
< 20%	82 (75)
≥ 20%	27 (25)
Necrosis	
Non-necrotic	70 (64)
5-30%	39 (36)
Inflammation	
< 20%	95 (87)
≥ 20%	14 (13)
Biopsy Type	
Fine needle aspirate (FNA)/ cell block	20 (18)
Core and other biopsies	52 (48)
Small excisions	37 (34)

#### 92-gene assay clinical validation

The 92-gene assay predicted a molecular diagnosis in 95% (N = 104) of cases (the remaining 5 cases were indeterminate) when compared to the gold standard of adjudicated histopathological diagnoses with integration of clinical findings and ancillary testing. The overall sensitivity in these cases was 91% [95% confidence interval (CI) 0.84 - 0.95; Figure 2C]. There was no difference in sensitivities between primary and metastatic cases (*p* = 0.73; Table 2). It was previously demonstrated in a large blinded validation study that the assay maintained high accuracy irrespective of specimen type (limited tissue vs non-limited tissue) [8]. In the current study, the performance of the 92-gene assay in the limited tissue subset was further analyzed and stratified based on biopsy types. Performance of the assay was consistent across biopsy types (FNA/cell blocks = 100%, core and other biopsies = 92%, small excisions = 86%; *p* = 0.26; Figure 2C) and across histologic grades (I = 100%, II = 90%, III = 89%, not graded = 93%; *p* = 0.84; Table 2). No statistical difference in performance was observed across graded tumors (histologic grades I, II and III; *p* = 0.75).

**Table 2:** 92-gene assay performance by clinical subset in the clinical validation cohort (N=109)

Clinical variables	N (%)	Sensitivity	*P*
Disease Type			0.73
Primary	57 (52)	93%	
Metastatic	52 (48)	90%	
Histologic Grade			0.84
Grade I	5 (5)	100%	
Grade II	20 (18)	90%	
Grade III	38 (35)	89%	
Not graded	46 (42)	93%	

### Analysis of cytology clinical case specimens

#### Distribution of biopsy sites and molecular diagnoses

**Figure 3:**
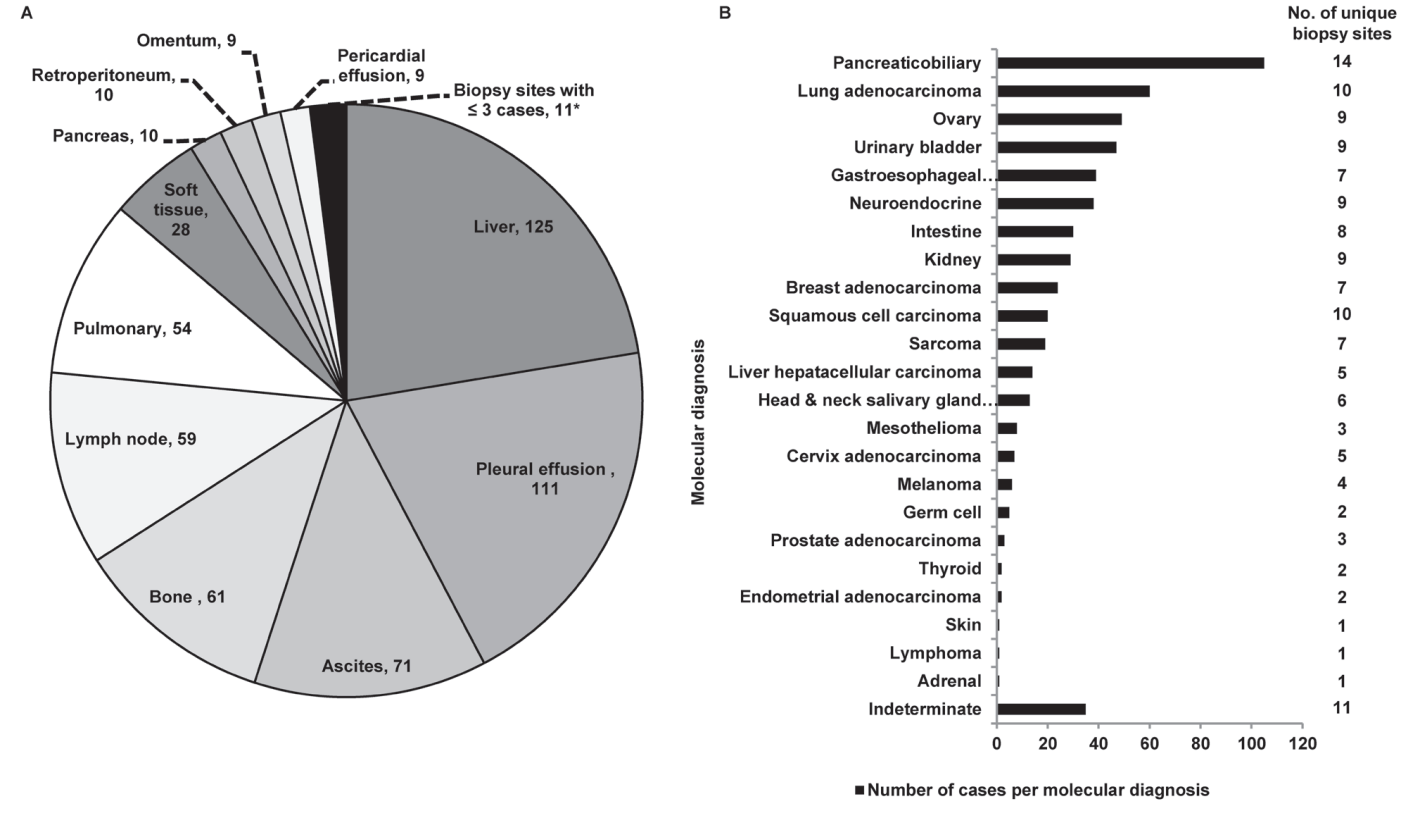
**A.** Distribution of biopsy sites within the clinical cytology case series that passed analytical QC (*N* = 558). *Biopsy sites with three or fewer cases were combined into a single category, which includes: Mediastinum (3), Bowel (2), Breast (2), Kidney (2), Adrenal (1), Periaortic (1), Thyroid (1). **B.** Distribution of molecular diagnoses as determined by the 92-gene assay in the clinical cytology data set that passed analytical QC (*N* = 558). Molecular diagnoses labeled on the y-axis. Number of unique biopsy sites for each molecular diagnosis is labeled to the right of the graph.

Of 644 cytology clinical cases submitted for testing with the 92-gene assay as part of routine clinical care, the 92-gene assay had an analytical success rate of 87%, while 13% of cases failed analytical QC due to insufficient RNA or poor RNA quality. Submitted cases were distributed among 18 biopsy sites with liver (n = 125), pleural effusion (n = 111), ascites (n = 71) and bone (n = 61) contributing to over 50% of the cases (Figure 3A). The 92-gene assay predicted 23 different tumor types; the four most common molecular diagnoses were pancreaticobiliary (19%; n = 105), lung adenocarcinoma (11%; n = 60), ovary (9%; n = 49) and urinary bladder (8%; n = 47) (Figure 3B). These four most common molecular diagnoses were detected across a large number of biopsy sites (pancreaticobiliary, n = 14; lung adenocarcinoma, n = 10; ovary, n = 9; and urinary bladder, n = 9; Figure 3B). Biopsies from the liver, pleural effusions, bone, lymph node, peritoneal effusions, lung, and soft tissue all had more than 10 different tumor types predicted by the 92-gene assay. Thirty-five cases (6%) were indeterminate, in which the assay did not report a probability of at least 70% for a single site diagnosis; however, the 92-gene assay test report for indeterminate cases does provide additional information that can help to establish a diagnosis, including a list of tumor types with some degree of gene expression overlap with known tumors within the reference database, as well as tumor types and subtypes that can be ruled out with a 95% confidence [8].

#### Site-specific Biomarker Testing

Thirty-seven cases with a 92-gene assay molecular diagnosis of lung (n = 22), colorectal (n = 6), gastric (n = 4), breast (n = 2), and melanoma (n = 3) from the clinical case dataset had ancillary biomarker testing performed from the same laboratory (Biotheranostics, Inc.) (Supplementary Table 1). Of these, 36 cases had successful biomarker testing. The most common biomarkers analyzed were *EGFR* mutations (n = 20), *KRAS* mutations (n = 12), *BRAF* mutation (n = 10), *PIK3CA* (n = 8) and *ALK* rearrangements (n *=* 8) (Supplementary Table 1). Within this group, 53% (n = 19) of cases had more than one predictive biomarker assessed. Of the cases predicted by the 92-gene assay to be lung and colorectal (n=28), *EGFR* and *KRAS* testing were performed in 18 and 5 cases, respectively. *EGFR* mutations were detected in 4 of the lung predictions and 4 of the colorectal predictions were identified to be wild type for *KRAS*.

In one case, a 64 year old female presented with malignant cells in a pleural effusion (Table 3). The pathology report indicated a diagnosis of adenocarcinoma with possible primary sites including the lung, pancreaticobiliary tract, and genital tract based on immunohistochemical stains. The 92-gene assay provided a molecular diagnosis of lung adenocarcinoma (probability = 96%), with all other suspected primary sites ruled out with 95% confidence. Predictive biomarker analysis detected a deletion of exon 19 in *EGFR*, while *ALK* rearrangement and *ROS-1* rearrangement were not detected (Table 3). These data indicate potential benefit from treatment with targeted molecular therapies. In another case, an 81 year old female presented with malignant cells in a pericardial effusion and was initially suspected to have a pancreaticobiliary or upper gastrointestinal malignancy (Table 4). The 92-gene assay resulted in a diagnosis of lung adenocarcinoma (probability = 90%) with an *EGFR* L858R mutation, enabling eligibility for EGFR targeted therapy options.

**Table 3:** Summary of the clinical characteristics, molecular diagnosis from 92-gene assay and results from predictive biomarker testing for an exemplar patient case from the clinical case cohort

Clinicopathologic characteristics		
Age	64 y	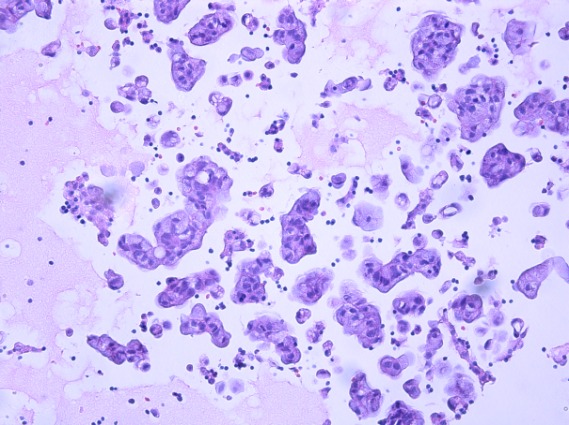
Gender	Female	
Biopsy site	Pleural effusion	
Pathology diagnosis	Adenocarcinoma	
Immunohistochemical analysis		
Cam5.2	Positive	
CK7	Positive	
MOC-31	Positive	
CA 19-9	Positive	
CA125	Positive	
TTF-1	Positive (weak)	
CK20	Negative	
Calretinin	Negative	
WT-1	Negative	
D2-40	Negative	
SP-A	Negative	
Napsin A	Negative	
CDX-2	Negative	
ER	Negative	
PR	Negative	
BRST-2	Negative	
Mammoglobin	Negative	
92-gene assay molecular diagnosis	Lung adenocarcinoma	
Biomarker analysis		
*EGFR* mutation	Exon 19 deletion	
*ALK* rearrangement	Not detected	
*ROS-1* rearrangement	Not detected	

**Table 4:** Summary of the clinical characteristics, molecular diagnosis from 92-gene assay and results from predictive biomarker testing from a clinical patient case utilizing the 92-gene assay and NGS testing

Clinicopathologic characteristics		
Age	81 y	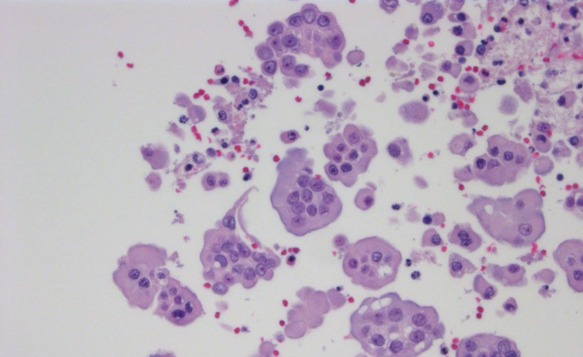
Gender	Female	
Biopsy site	Pericardial fluid	
Pathology diagnosis	Pancreaticobiliary vs Upper GI	
Immunohistochemical analysis		
CEA	Positive	
CK7	Positive	
MUC4	Positive (weak)	
CK5/6	Positive (weak)	
TTF-1	Negative	
Napsin A	Negative	
CDX-2	Negative	
CK20	Negative	
ER	Negative	
PAX-8	Negative	
GATA-3	Negative	
Calretinin	Negative	
92-gene assay molecular diagnosis	Lung adenocarcinoma	
Biomarker analysis		
*EGFR* mutation	Exon 21 L858R	
*KIT* mutation	Exon 10 M541L	
*TP53* mutation	R273S	
*ALK* rearrangement	Not detected	
*ROS-1* rearrangement	Not detected	
*RET* rearrangement	Not detected	
PD-L1 expression	Not detected	

## DISCUSSION

Advances in genomics and cancer-targeting technologies have provided new diagnostic and treatment options for an increasing range of metastatic malignancies [16, 17]. In cases where small amounts of tissue are obtained from minimally invasive biopsies, the extent of diagnostic work-up for accurate diagnosis and the technical success of genomic assays that now are often recommended to define targeted treatment options may be limited [18]. Thoughtful use of complementary techniques such as IHC, fluorescence in situ hybridization (FISH), mutational and gene expression-based analyses is critical for optimizing disease management in oncology. Implementation of a tissue-sparing platform, such as the 92-gene assay, provides a standardized approach for tumor classification in cases wherein limited biopsy specimen is available.

In addition to the 92-gene assay, several validated molecular cancer classifiers that analyze either messenger RNA or microRNA are commercially available [8, 9, 19]. Prior studies of microarray-based classifiers have previously assessed feasibility in a small cohort of cytologic specimens (n = 27), however, blinded validation of performance was not conducted [20]. Findings from this study show that the 92-gene assay demonstrated high sensitivity (91%) in the blinded validation of the limited tissue cohort (Figure 2C). Importantly, the accuracy of the assay was maintained across specimen types (Figure 2C), clinically relevant subsets (histologic grade and disease type) (Table 2), and is comparable to the accuracy observed in non-limited specimens (excisions and resections) from the previously published 790-sample blinded validation of the 92-gene assay [8].

Examination of the analytical success rate of the 92-gene assay in the consecutive clinical cytology cases illustrated high success rate (87%), irrespective of lower tumor content and cellularity than required by the assay based on histopathological examination. The 92-gene assay distinguished multiple tumor types from a range of biopsy sites (Figures 2A, 2B, and 3). The observed spectrum of molecular diagnoses in the cytology clinical case series (23 distinct tumor types) highlights the difficulty in establishing a diagnosis and treatment plan when analyzing metastatic and poorly differentiated tumors from a limited tissue specimen. A key contributing factor to the analytical success of the 92-gene assay is its ability to interrogate multiple biologic pathways while consuming a minimal amount of tissue from specimens with a range of cellularity [8, 12]. Furthermore, the 92-gene assay has been optimized to manage additional sample complexity arising from tissue preservation methodologies like the use of FFPE samples, which can cause degradation of the nucleic acid content within the specimen.

Utilization of tissue sparing technologies can impact downstream analyses on patient tissue samples. While not the objective of this study, the 92-gene assay in the clinical case series allowed for additional biomarker or mutation analysis of actionable targets within a tumor type. Thirty-six of the 37 cytology cases with a diagnosis of lung-, colorectal-, gastric-, breast adenocarcinoma, and melanoma had additional biomarker testing performed at the same lab (Supplementary Table 1). These additional analyses can provide useful information with respect to treatment planning and individualized response to therapy (Table 3 and Table 4). These data illustrate that molecular techniques such as the 92-gene assay may allow combined diagnostic and biomarker testing for limited tissue specimens to maximize their diagnostic yield (Supplementary Table 1).

New techniques such as NGS provide an increasing amount of information while decreasing tissue consumption, however, knowledge of histology and tumor type continue to be critical for individualizing treatment. Growing understanding of cancer biology has led to an emerging form of clinical trial design, termed “Basket Trials,” based on the hypothesis that a molecular alteration predicts response to a targeted therapy independent of tumor histology. Initial results from these studies have shown that integration of tumor type/cellular context continues to be fundamental in the era of precision medicine and one cannot extrapolate the effectiveness of a targeted therapy uniformly to all cancer types with a matching molecular alteration [21, 22]. These data underscore the importance of having an accurate diagnosis for metastatic cancer patients to enable responsible precision medicine. For example, in both case studies presented, the molecular diagnosis from the 92-gene assay in conjunction with downstream biomarker analyses provided actionable information including eligibility and efficacy for specific targeted therapies and clinical trial protocols that can be used to tailor each patient's treatment regimen.

**Figure 4:**
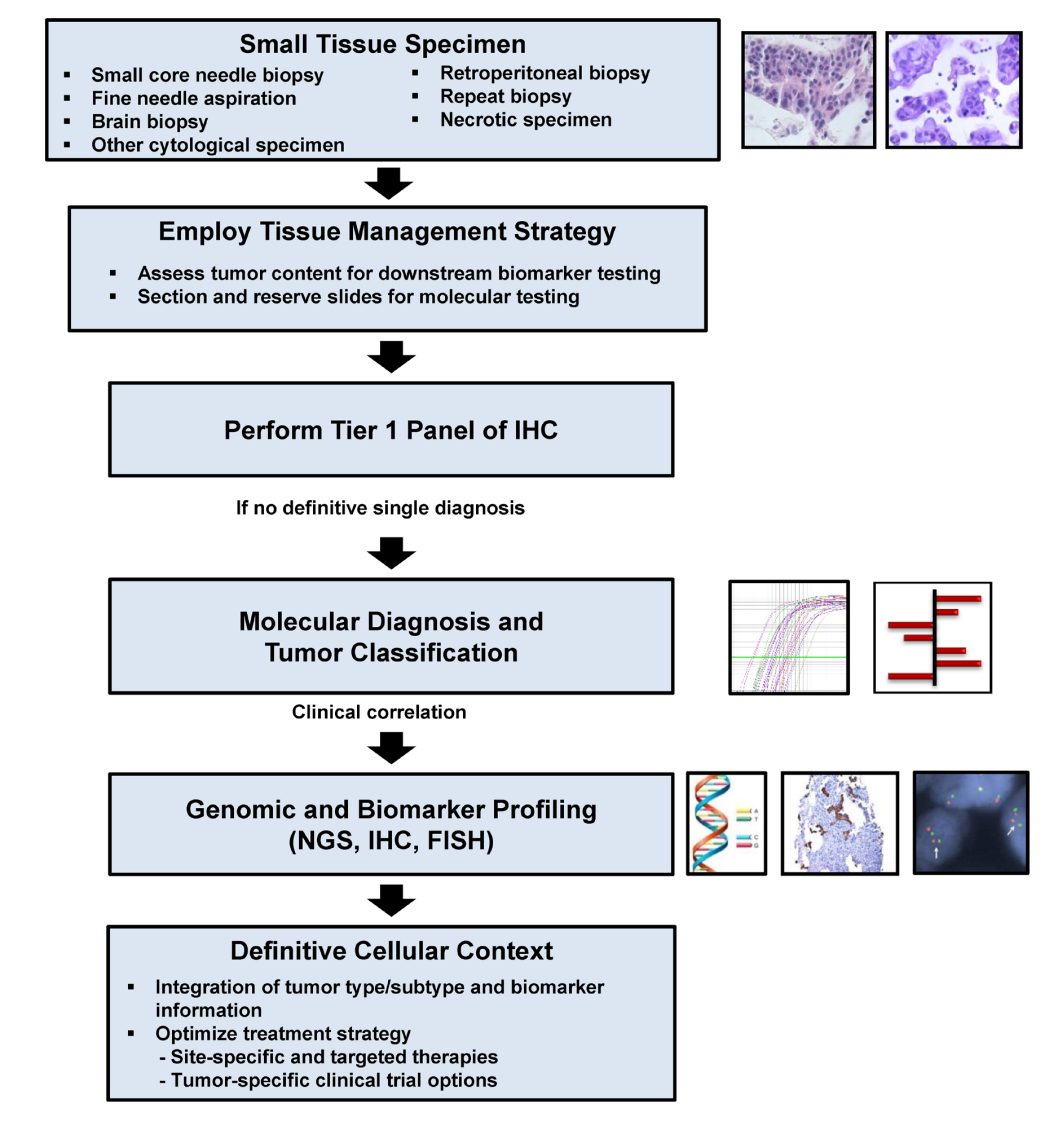
Proposed tissue-based diagnostic algorithm Adapted from (Schnabel & Erlander, Expert Opin. Med. Diag. 2012). Abbreviations: IHC = Immunohistochemistry, FISH = Fluorescence in-situ hybridization, NGS = Next generation sequencing.

In addition to providing a diagnosis, pathologists are responsible for the strategic use of tissue specimens in molecular studies [23–25]. Use of a tissue-based, diagnostic algorithm which incorporates molecular testing may help preserve the tissue specimen and allow for further evaluation (Figure 4). Diagnostic algorithms should be flexible according to individual clinical and pathological characteristics (*e.g.* working diagnosis, histologic appearance, presence of metastases, and tumor location), as well as the needs and case distributions at a clinical practice (Figure 4). Clinicians and pathologists, as members of an oncology care team may implement tissue-sparing diagnostic algorithms based on consensus decisions custom-tailored to their patient population. For example, rapid interpretation of FNA slides is a means to ensure adequate tissue sampling while the diagnostic procedure is in progress; this requires trained personnel and can be done on-site in radiology or endoscopy suites or remotely with the help of digital imaging technology [26, 27]. Quality control mechanisms and appropriate handling are needed to ensure tissue sample preservation and avoid exhaustion of blocks [28]. Additionally, effective reporting mechanisms have to be in place to communicate not only the diagnosis but also quality and quantity of tumor cells in any given tissue sample [5]. The goal of combining these various sampling and analytic approaches is to ensure accurate diagnosis and allocation of tissue from small samples for the appropriate ancillary studies at the point of care.

Key findings of this study demonstrate the high accuracy and analytical success rate of the 92-gene assay in limited tissue samples, providing further support of the diagnostic utility of the 92-gene assay for cytology and limited tissue samples. A limitation of the study was that although a high analytical success rate was observed in clinical cytology specimens, clinical validation by definitive follow up could not be performed at this point. Further studies to include clinical follow up and patient consent are needed to define these parameters in more detail. Additionally, further studies assessing the economic impact, effect on time to treatment, and patient outcomes after molecular diagnosis are merited. Incorporation of molecular classifiers such as the 92-gene assay that have been designed as a tissue sparing technology may serve as a safeguard for tissue preservation, especially in those cases where predictive biomarker analysis is warranted, and mitigate the risks associated with repeated biopsy procedures.

## MATERIALS AND METHODS

Patient cases that were examined in this report included 1) samples from a prospective blinded clinical validation and 2) a case series from clinical testing.

### Blinded clinical validation

A validation cohort consisting of N=109 limited tissue samples that were part of a larger 790-sample blinded validation were evaluated. Details of case selection, adjudication, sample processing and analysis of the samples for the validation cohort (N = 109) were previously described [8]. In brief, formalin-fixed paraffin-embedded (FFPE) tissue samples of known specimen type and histopathologic diagnoses that met study inclusion criteria were selected with waived patient consent by the respective IRBs. Representative slides were scanned, uploaded to a digital pathology platform (Aperio^®^), and diagnostically adjudicated. Definitive histopathological diagnoses (gold standard) were established utilizing ancillary tests and integration of clinical, radiographic and follow-up information, and where consensus was obtained during adjudication by at least two board certified study pathologists. Samples were categorized as “limited” in the parent study based on specimen type and confirmed by microscopic review by the adjudicating pathologists. Limited tissue samples were defined as cell blocks from fine needle aspiration (FNA) samples (N = 20), core biopsies and other small biopsy specimens such as skin punch or shave biopsies and curettings (N = 52), and small excisions with tumor spanning less than 1 cm (N = 37), while non-limited tissue samples were from surgical procedures where entire tumors were available for analysis. Inclusion criteria were specified in the protocol of the parent study; (i) tumors processed less than 6 years prior to testing, (ii) diagnosis contained within the assay panel, (iii) at least 40% tumor available in a markable area on the hematoxylin and eosin (H&E) slide, (iv) minimal necrosis, and (v) result within the analytical QC requirement of the assay.

### Clinical case series

The 92-gene assay was performed on 644 consecutive cytology samples submitted as part of routine clinical evaluation. The study was approved by Western Institutional Review Board (WIRB) with waived individual patient consent. Selection criteria for clinical cytology cases included any FNA biopsy or cell block containing cytologic body fluid specimens (pleural effusions, ascites, and pericardial effusions). In cytology cases where the number of tumor cells was lower than the suggested minimum requirements for the 92-gene assay, the assay was performed with fewer tumor cells at the discretion of the reviewing pathologist, and results reported for cases that did not exceed the PCR analytical cutoff value for internal controls (cycling threshold > 29). Additionally, biomarker analysis (*EGFR*, *KRAS*, *BRAF*, *PIK3CA*, and *ALK* rearrangements) was performed for cases in which the clinician requested biomarker testing subsequent to the 92-gene assay results of lung-, colorectal-, gastric-, and breast carcinoma and melanoma.

### 92-Gene assay

Cases were analyzed and molecular diagnosis predicted using the 92-gene assay (CancerTYPE ID^®^, Biotheranostics, Inc.) as described previously [8, 12]. Tumor cells were captured and enriched via laser microdissection (LMD) and RNA extracted from the specimen. Real-time RT-PCR analysis yields a collective gene expression profile from 87 tumor-related genes and 5 internal reference genes. An algorithm compares the patient's gene expression profile to expression profiles from a reference database composed of known tumor types thereby generating a molecular diagnosis reported as a percent probability [8, 12]. Input requirements for the 92-gene assay include approximately 300 to 500 non-necrotic tumor cells. Sample input range is generally between 0.2 nanograms to 200 nanograms of RNA. Cases exceeding the PCR analytical cut-off for internal controls (cycling threshold (C_t_) >29) were considered quality control failures.

### Data analyses

Overall sensitivity (i.e., diagnostic accuracy) of the 92-gene assay was calculated by comparing the molecular diagnosis to the definitive histopathological diagnosis obtained by consensus adjudication by two board certified pathologists who integrated morphology, ancillary testing, clinical and radiologic findings during the validation study (gold standard). Number of concordant cases was then divided by the total number of cases classifiable by the assay. Cases were categorized as unclassifiable or indeterminate if the algorithm did not report a probability of at least 70% for a single site diagnosis. Specificity was calculated as the number of true negative results divided by the sum of true negatives and false positives. Both sensitivity and specificity calculations employed a two-sided 95% confidence interval. Fisher's exact test was used to compare diagnostic accuracy between clinical subsets. The performance of the 92-gene assay in the limited tissue subset (N = 109) was further examined according to biopsy type (FNA/ cell block, core biopsy, other small biopsies and small excisions). Analysis of the performance of the 92-gene assay with respect to disease type and histologic grade was also conducted. Analytical success rate of the 92-gene assay for the clinical samples was defined as the proportion of cases passing pathology review, analytical quality control, and completion of 92-gene assay testing.

## SUPPLEMENTARY MATERIALS TABLES



## References

[1] Heitzer E, Ulz P, Geigl JB (2015). Circulating tumor DNA as a liquid biopsy for cancer. Clin Chem.

[2] Ilie M, Hofman V, Long E, Bordone O, Selva E, Washetine K, Marquette CH, Hofman P (2014). Current challenges for detection of circulating tumor cells and cell-free circulating nucleic acids, and their characterization in non-small cell lung carcinoma patients. What is the best blood substrate for personalized medicine?. Ann Transl Med.

[3] Dumur CI, Idowu MO, Powers CN (2013). Targeting tyrosine kinases in cancer: the converging roles of cytopathology and molecular pathology in the era of genomic medicine. Cancer Cytopathol.

[4] Koboldt DC, Steinberg KM, Larson DE, Wilson RK, Mardis ER (2013). The next-generation sequencing revolution and its impact on genomics. Cell.

[5] Cagle PT, Sholl LM, Lindeman NI, Alsabeh R, Divaris DX, Foulis P, Lee G, Neal JW, Nowak JA, Yu PP, Members of the Cancer Biomarker Reporting Workgroup CoAP (2014). Template for reporting results of biomarker testing of specimens from patients with non-small cell carcinoma of the lung. Arch Pathol Lab Med.

[6] Rafael OC, Aziz M, Raftopoulos H, Vele OE, Xu W, Sugrue C (2014). Molecular testing in lung cancer: fine-needle aspiration specimen adequacy and test prioritization prior to the CAP/IASLC/AMP Molecular Testing Guideline publication. Cancer Cytopathol.

[7] Hur J, Lee HJ, Nam JE, Kim YJ, Hong YJ, Kim HY, Kim SK, Chang J, Kim JH, Chung KY, Lee HS, Choi BW (2012). Additional diagnostic value of tumor markers in cytological fluid for diagnosis of non-small-cell lung cancer. BMC Cancer.

[8] Kerr SE, Schnabel CA, Sullivan PS, Zhang Y, Singh V, Carey B, Erlander MG, Highsmith WE, Dry SM, Brachtel EF (2012). Multisite validation study to determine performance characteristics of a 92-gene molecular cancer classifier. Clin Cancer Res.

[9] Pillai R, Deeter R, Rigl CT, Nystrom JS, Miller MH, Buturovic L, Henner WD (2011). Validation and reproducibility of a microarray-based gene expression test for tumor identification in formalin-fixed, paraffin-embedded specimens. J Mol Diagn.

[10] Sokilde R, Vincent M, Moller AK, Hansen A, Hoiby PE, Blondal T, Nielsen BS, Daugaard G, Moller S, Litman T (2014). Efficient identification of miRNAs for classification of tumor origin. J Mol Diagn.

[11] Varadhachary GR, Raber MN (2014). Carcinoma of unknown primary site. N Engl J Med.

[12] Erlander MG, Ma XJ, Kesty NC, Bao L, Salunga R, Schnabel CA (2011). Performance and clinical evaluation of the 92-gene real-time PCR assay for tumor classification. J Mol Diagn.

[13] Ma XJ, Patel R, Wang X, Salunga R, Murage J, Desai R, Tuggle JT, Wang W, Chu S, Stecker K, Raja R, Robin H, Moore M (2006). Molecular classification of human cancers using a 92-gene real-time quantitative polymerase chain reaction assay. Arch Pathol Lab Med.

[14] Weiss LM, Chu P, Schroeder BE, Singh V, Zhang Y, Erlander MG, Schnabel CA (2013). Blinded comparator study of immunohistochemical analysis versus a 92-gene cancer classifier in the diagnosis of the primary site in metastatic tumors. J Mol Diagn.

[15] Hainsworth JD, Rubin MS, Spigel DR, Boccia RV, Raby S, Quinn R, Greco FA (2013). Molecular gene expression profiling to predict the tissue of origin and direct site-specific therapy in patients with carcinoma of unknown primary site: a prospective trial of the Sarah Cannon research institute. J Clin Oncol.

[16] Larkin J, Ascierto PA, Dreno B, Atkinson V, Liszkay G, Maio M, Mandala M, Demidov L, Stroyakovskiy D, Thomas L, de la Cruz-Merino L, Dutriaux C, Garbe C (2014). Combined vemurafenib and cobimetinib in BRAF-mutated melanoma. N Engl J Med.

[17] Shaw AT, Engelman JA (2014). Ceritinib in ALK-rearranged non-small-cell lung cancer. N Engl J Med.

[18] Karnes HE, Duncavage EJ, Bernadt CT (2014). Targeted next-generation sequencing using fine-needle aspirates from adenocarcinomas of the lung. Cancer Cytopathol.

[19] Rosenwald S, Gilad S, Benjamin S, Lebanony D, Dromi N, Faerman A, Benjamin H, Tamir R, Ezagouri M, Goren E, Barshack I, Nass D, Tobar A (2010). Validation of a microRNA-based qRT-PCR test for accurate identification of tumor tissue origin. Mod Pathol.

[20] Stancel GA, Coffey D, Alvarez K, Halks-Miller M, Lal A, Mody D, Koen T, Fairley T, Monzon FA (2012). Identification of tissue of origin in body fluid specimens using a gene expression microarray assay. Cancer Cytopathol.

[21] Hyman DM, Puzanov I, Subbiah V, Faris JE, Chau I, Blay JY, Wolf J, Raje NS, Diamond EL, Hollebecque A, Gervais R, Elez-Fernandez ME, Italiano A (2015). Vemurafenib in Multiple Nonmelanoma Cancers with BRAF V600 Mutations. N Engl J Med.

[22] Le Tourneau C, Delord JP, Goncalves A, Gavoille C, Dubot C, Isambert N, Campone M, Tredan O, Massiani MA, Mauborgne C, Armanet S, Servant N, Bieche I (2015). Molecularly targeted therapy based on tumour molecular profiling versus conventional therapy for advanced cancer (SHIVA): a multicentre, open-label, proof-of-concept, randomised, controlled phase 2 trial. Lancet Oncol.

[23] Cagle PT, Allen TC, Olsen RJ (2013). Lung cancer biomarkers: present status and future developments. Arch Pathol Lab Med.

[24] Schnabel CA, Erlander MG (2012). Gene expression-based diagnostics for molecular cancer classification of difficult to diagnose tumors. Expert Opin Med Diagn.

[25] Travis WD, Brambilla E, Noguchi M, Nicholson AG, Geisinger K, Yatabe Y, Ishikawa Y, Wistuba I, Flieder DB, Franklin W, Gazdar A, Hasleton PS, Henderson DW (2013). Diagnosis of lung cancer in small biopsies and cytology: implications of the 2011 International Association for the Study of Lung Cancer/American Thoracic Society/European Respiratory Society classification. Arch Pathol Lab Med.

[26] da Cunha Santos G, Boerner SL, Geddie WR (2011). Maximizing the yield of lymph node cytology: Lessons learned from rapid onsite evaluation of image- and endoscopic-guided biopsies of hilar and mediastinal lymph nodes. Cancer Cytopathol.

[27] Schmidt RL, Witt BL, Lopez-Calderon LE, Layfield LJ (2013). The influence of rapid onsite evaluation on the adequacy rate of fine-needle aspiration cytology: a systematic review and meta-analysis. Am J Clin Pathol.

[28] Kapp JR, Diss T, Spicer J, Gandy M, Schrijver I, Jennings LJ, Li MM, Tsongalis GJ, de Castro DG, Bridge JA, Wallace A, Deignan JL, Hing S (2015). Variation in pre-PCR processing of FFPE samples leads to discrepancies in BRAF and EGFR mutation detection: a diagnostic RING trial. J Clin Pathol.

