# Supplementary material for: Molecular classification of cancer with the 92-gene assay in cytology and limited tissue samples
Source: Oncotarget. 2016 Mar 28;7(19):27220–31. doi: 10.18632/oncotarget.8449 (PMC5053644; doi:10.18632/oncotarget.8449)
Supplement: Supplementary file 1 [file oncotarget-07-27220-s001.pdf]

Molecular classification of cancer with the 92-gene assay in cytology and limited tissue samples

Supplementary Material

Supplementary Table 1: Utilization of the 92-gene assay in the clinical case cohort for establishing a molecular diagnosis and directing predictive biomarker analysis

**Case characteristics**	***N***
Number of cases where biomarker testing was performed*	
(lung, colorectal, gastric, breast, melanoma)	37
Cases that had biomarker analysis performed	36
≥ 2 Biomarkers assessed	19
Most common biomarkers assessed	
EGFR	20
KRAS	12
BRAF	10
PIK3CA	8
ALK rearrangement	8

*Biomarker analysis had to be performed at the same lab as the 92-gene assay to be included in this table
